# Comparative Prognostic Performance of qSOFA and SOFA in Emergency Department Patients With Established Septic Shock

**DOI:** 10.1155/emmi/1902660

**Published:** 2026-08-27

**Authors:** Xue Liu, Huajie Zhang, Qirong He

**Affiliations:** ^1^ Emergency Medicine Department, Yibin First People’s Hospital, No. 16 Puhe East Road Xuzhou Avenue Xuzhou District, Yibin Sichuan, 644000, China, scu.edu.cn; ^2^ Third Department of Tuberculosis, Kunming Third People’s Hospital, No. 318 Wujing Road Guandu District, Kunming Yunnan, 650000, China; ^3^ Comprehensive Internal Medicine Department, Yunnan Provincial Infectious Diseases Hospital, 28 km Mark on Shi’an Highway, Anning Yunnan, 650300, China

**Keywords:** emergency department, mortality prediction, prognostic assessment, qSOFA, septic shock, SOFA

## Abstract

**Background:**

The quick sequential organ failure assessment (qSOFA) enables rapid bedside screening, whereas sequential organ failure assessment (SOFA) offers a more comprehensive evaluation of organ dysfunction; their comparative prognostic value in established septic shock remains unclear.

**Objective:**

To compare the prognostic performance of qSOFA and SOFA for 28‐day mortality prediction in patients with established septic shock according to Sepsis‐3 criteria presenting to the emergency department and to evaluate the applicability of a simplified bedside scoring tool (qSOFA) within this specific high‐risk population.

**Methods:**

This retrospective cohort study included 486 patients with complete qSOFA and SOFA data from January 2021 to December 2024. Receiver operating characteristic curves, DeLong tests, multivariable logistic regression, Kaplan–Meier analysis, and calibration testing were performed. Multiple imputation of missing SOFA components was applied in a sensitivity analysis comprising 527 clinically eligible patients, and exploratory subgroup analyses were conducted according to infection source.

**Results:**

The 28‐day mortality rate was 38.5%. SOFA provided better mortality discrimination than qSOFA (AUC 0.847, 95% CI 0.812–0.882 vs. 0.724, 95% CI 0.679–0.769; *p* < 0.001) and also outperformed qSOFA in predicting ICU admission (AUC 0.798 vs. 0.686; *p* < 0.001). The SOFA score, age, and lactate level emerged as independent predictors of mortality. The addition of lactate improved qSOFA performance (AUC 0.789), and the findings remained robust in multiple‐imputation and infection‐source subgroup analyses.

**Conclusion:**

In patients with established septic shock according to Sepsis‐3 criteria, SOFA outperformed qSOFA in mortality risk stratification. qSOFA may be useful for rapid bedside screening but should not replace the comprehensive prognostic assessment provided by SOFA.

## 1. Introduction

Global healthcare systems face an enormous burden from sepsis and septic shock. According to recent epidemiological analyses, these conditions account for approximately 48.9 million cases and 11 million deaths annually, representing roughly one‐fifth of total global mortality [1]. Septic shock is defined by circulatory collapse, cellular dysfunction, and metabolic derangements that require vasopressor support to maintain mean arterial pressure above 65 mmHg, along with lactate levels exceeding 2 mmol/L despite adequate fluid resuscitation. Even in optimally resourced facilities, mortality rates range from 40% to 60% [2]. A fundamental clinical challenge is that most patients with septic shock initially present through emergency departments, making this setting the critical determinant of treatment timing and patient outcomes [3]. Each hour of delay in antimicrobial administration has been shown to correlate with a stepwise increase in mortality [4]. Consequently, the development and validation of rapid assessment instruments for emergency department use have become a research and clinical priority of paramount importance.

The conceptual foundation of contemporary sepsis severity assessment rests on the sequential organ failure assessment (SOFA) score, originally formulated by the European Society of Intensive Care Medicine in 1994 and later integrated into the Sepsis‐3 consensus definitions [5]. SOFA evaluates dysfunction across six organ systems—respiratory, cardiovascular, hepatic, coagulation, renal, and neurological—and generates a composite score ranging from 0 to 24, which demonstrates robust correlation with mortality [6]. Current sepsis definitions incorporate an acute increase in SOFA of 2 or more points as a defining criterion. Nevertheless, a full SOFA calculation requires laboratory parameters—arterial blood gases, bilirubin, platelet count, and creatinine—that may not be immediately available upon emergency department arrival, potentially limiting its utility for initial rapid assessment [7]. The computational complexity and need for baseline values pose additional practical challenges in settings where rapid clinical decision‐making is essential.

Given these constraints, the Sepsis‐3 Task Force introduced the quick SOFA (qSOFA) as a simplified bedside screening tool designed to identify patients with suspected infection who are at increased risk of adverse outcomes [8]. qSOFA comprises three readily assessable clinical variables: respiratory rate ≥ 22 breaths/min, altered mental status (Glasgow Coma Scale < 15), and systolic blood pressure ≤ 100 mmHg—each contributing one point toward a maximum score of 3 [9]. The main appeal of qSOFA lies in its simplicity: It requires no laboratory testing or complex calculations, enabling immediate bedside assessment by clinicians of any experience level. Initial validation studies by Shankar‐Hari et al. suggested that qSOFA provided mortality prediction comparable to SOFA outside intensive care settings, facilitating its endorsement in the Sepsis‐3 guidelines [10]. However, subsequent investigations, including those by Fernando et al., have generated conflicting evidence regarding the diagnostic performance of qSOFA, with multiple studies reporting concerning sensitivity deficits for sepsis identification that could lead to missed or delayed diagnoses [11].

The comparative performance of qSOFA versus SOFA in emergency department patients with established septic shock according to Sepsis‐3 criteria—as distinct from broader sepsis cohorts—has received limited systematic investigation, representing a knowledge gap with direct clinical implications [12]. Sabir et al. noted that emergency department septic shock patients constitute a distinct population characterized by advanced disease severity, higher acuity, and compressed decision‐making timeframes compared with the general ward populations in which much of the qSOFA validation literature originated [13]. Emergency department environments present unique challenges—limited access to historical information, variable availability of laboratory resources, and the need for rapid triage—that may differentially affect the utility of each scoring system [14]. Furthermore, Finkelsztein et al. demonstrated that prognostic accuracy may vary across septic shock phenotypes and infection sources, underscoring the need for population‐specific validation [15].

Beyond mortality prediction, the comparative utility of qSOFA and SOFA for predicting clinically relevant secondary outcomes—including intensive care unit (ICU) admission, vasopressor requirements, mechanical ventilation, and length of hospital stay—remains incompletely characterized in emergency department septic shock populations. Clarifying predictive performance across multiple outcome domains would help guide optimal integration of these scores into emergency department workflows. In addition, whether combining qSOFA with readily available biomarkers such as lactate could enhance predictive accuracy warrants rigorous evaluation as a practical clinical strategy.

The present study aimed to compare the mortality risk‐stratification performance of qSOFA and SOFA in emergency department patients with established septic shock according to Sepsis‐3 criteria and to assess the clinical applicability of qSOFA in this specific high‐risk population. We hypothesized that SOFA would show superior 28‐day mortality prediction owing to its comprehensive organ dysfunction assessment, while qSOFA might remain useful as a rapid initial screening tool. The primary objective was to compare the AUCs for 28‐day mortality prediction; secondary objectives were to assess ICU admission prediction accuracy and to determine whether the addition of lactate could improve qSOFA performance.

## 2. Methodology

### 2.1. Study Design and Setting

This retrospective cohort investigation was conducted within the emergency department of Yibin First People’s Hospital, a tertiary academic medical center with annual emergency department volume exceeding 72,000 visits. The observation period extended from January 1, 2021, through December 31, 2024. Institutional Review Board approval was obtained from Yibin First People’s Hospital (Approval number: SYBFL1250551, with approved date 2025/12/25), with informed consent requirements waived given the retrospective design. All procedures adhered to Declaration of Helsinki principles. The study was conducted and reported in accordance with the STROBE statement for observational studies, ensuring transparency in reporting of participant selection, missing data handling, and statistical analysis. In addition, the PROBAST (Prediction model Risk Of Bias Assessment Tool) framework was applied to evaluate the risk of bias and applicability of the prediction models. According to the PROBAST assessment, the analysis domain showed low risk of bias due to appropriate model construction and validation procedures (Supporting Figure 1). However, the participants’ domain demonstrated moderate risk because of the single‐center retrospective design.

Both STROBE and TRIPOD reporting standards were strictly followed to ensure transparency and reproducibility of the study.

Model development and performance reporting followed the TRIPOD (Transparent Reporting of a multivariable prediction model for Individual Prognosis Or Diagnosis) guidelines.

The assessment evaluates four domains: participants, predictors, outcome, and analysis. The participant domain showed moderate risk of bias due to the retrospective single‐center design, while the predictor, outcome, and analysis domains were rated as low risk. Overall, the model demonstrated acceptable methodological quality with limited risk of bias affecting internal validity.

### 2.2. Study Population

Patients were identified through systematic electronic medical record review using International Classification of Diseases, Tenth Revision (ICD‐10) code for septic shock (R65.21). The following inclusion criteria were applied: (1) age ≥ 18 years; (2) presentation to the emergency department with suspected or confirmed infection; and (3) diagnosis of septic shock according to Sepsis‐3 criteria (persistent hypotension requiring vasopressors to maintain mean arterial pressure ≥ 65 mmHg, along with serum lactate > 2 mmol/L despite adequate volume resuscitation); and (4) complete data availability allowing calculation of both qSOFA and SOFA at presentation.

Exclusion criteria were (1) age < 18 years; (2) pre‐arrival cardiac arrest; (3) advance directives limiting resuscitative interventions; (4) transfer from another acute care facility after > 24 h of prior treatment; (5) incomplete records preventing score calculation; (6) pregnancy; and (7) documented do‐not‐resuscitate status at presentation.

Sample size was determined assuming a comparison of two correlated ROC curves. Based on anticipated AUCs of 0.75 for qSOFA and 0.85 for SOFA derived from prior literature, with *α* = 0.05, power = 0.80, and an inter‐score correlation of 0.5, the minimum required enrollment was 420 patients. Accounting for a potential 15% rate of incomplete data, we targeted approximately 480 patients.

### 2.3. Data Collection and Variables

Two trained research coordinators extracted data using standardized case report forms. Discrepancies were resolved through consensus review by a senior investigator. The following variables were collected: demographics (age, sex, body mass index); comorbidities (diabetes mellitus, hypertension, chronic kidney disease, chronic obstructive pulmonary disease, malignancy, immunosuppression); infection source (pulmonary, abdominal, urinary, soft tissue, bloodstream, other/unknown); vital signs at presentation (heart rate, respiratory rate, systolic/diastolic blood pressure, temperature, oxygen saturation, Glasgow Coma Scale); laboratory values (white blood cell count, hemoglobin, platelet count, creatinine, bilirubin, lactate, procalcitonin, C‐reactive protein, arterial blood gas parameters); and treatment variables (time to antibiotic administration, vasopressor use, mechanical ventilation, fluid resuscitation volume).

Variable definitions and assessment windows: Consciousness was assessed via the Glasgow Coma Scale; mean arterial pressure and vasopressor dose were recorded; FiO_2_ and urine output were collected for the first 24 h; laboratory variables (creatinine, bilirubin, platelet, lactate, and PaO_2_/FiO_2_) were obtained at emergency department presentation only (index time point) in order to ensure consistency with real‐time emergency risk stratification and avoid information leakage from post‐admission data; the infection source was classified as pulmonary, abdominal, urinary, soft tissue, bloodstream, or other/unknown; outcomes included 28‐day and in‐hospital mortality. Missingness was assessed for each clinical and laboratory variable required for SOFA calculation. The primary analysis was based on 486 patients with complete data for both SOFA and qSOFA calculations. Forty‐one patients with one or more missing SOFA components were excluded from the primary complete‐case analysis. Because missingness was associated with higher lactate levels, these patients likely represented a more severely ill subgroup. Their exclusion may therefore have introduced selection bias, leading to underestimation of mortality risk and model discrimination. Overall, a complete‐case approach may disproportionately exclude sicker patients with incomplete laboratory assessments in emergency settings, potentially biasing both effect estimates and model performance toward more stable clinical presentations. This approach may introduce selection bias, as patients with incomplete laboratory data appeared more clinically severe, as reflected by higher lactate levels, and their exclusion may therefore lead to underestimation of mortality risk and potential bias in model performance estimates. To address this limitation, these patients were included in a multiple‐imputation sensitivity analysis, resulting in a total sensitivity cohort of 527 patients. Multiple imputation was used to reduce selection bias and improve the robustness of the findings.

Detailed variable definitions, measurement times, data sources, and missing data handling are provided in Supporting Table 1.

PaO_2_/FiO_2_ estimation procedure: In patients lacking arterial blood gas measurements, PaO_2_/FiO_2_ was estimated using SpO_2_/FiO_2_ according to Rice et al. [16]. Patients with SpO_2_ outside 80%–97% or evidence of poor peripheral perfusion were excluded from estimation. This affected 78 patients (16.1% of total), with 42 survivors and 36 nonsurvivors. Sensitivity analyses restricted to measured PaO_2_ confirmed similar SOFA performance.

### 2.4. Scoring Systems

#### 2.4.1. Quick SOFA (qSOFA) Score

The qSOFA computation utilized three clinical parameters assessed at emergency department presentation and during the first 24 h if multiple readings were available: respiratory rate ≥ 22 breaths/minute (1 point), altered mentation defined as Glasgow Coma Scale < 15 (1 point), and systolic blood pressure ≤ 100 mmHg (1 point). Total scores ranged from 0 to 3, with ≥ 2 considered positive for elevated mortality risk per Sepsis‐3 recommendations. For all scoring systems, variables were calculated based on values recorded at emergency department presentation (index time point). No post‐admission or worst‐within‐24‐h values were used to avoid inflation of predictive performance and to ensure clinical applicability at the time of initial triage. qSOFA requires no laboratory testing and can be calculated within seconds, facilitating rapid bedside screening.

#### 2.4.2. SOFA Score

SOFA scores were calculated using six organ system components measured at emergency department presentation and within the first 24 h of admission. The components included respiratory (PaO_2_/FiO_2_ ratio, 0–4 points), coagulation (platelet count, 0–4 points), hepatic (bilirubin, 0–4 points), cardiovascular (mean arterial pressure and vasopressor dose, 0–4 points), central nervous system (Glasgow Coma Scale, 0–4 points), and renal (creatinine or urine output, 0–4 points). For variables with multiple measurements during this window, the worst value was used. For patients without arterial blood gas (ABG) measurements, the PaO_2_/FiO_2_ ratio was estimated from SpO_2_/FiO_2_ using the validated conversion equation proposed by Rice et al. [16]:

(1) PaO2FiO2=640.84+×SpO2FiO2.

This approach was applied to 78 patients (16.1% of the study cohort), including 42 survivors and 36 nonsurvivors. Estimation was restricted to patients with SpO_2_ between 80% and 97%, and patients with SpO_2_ values outside this range or with evidence of poor peripheral perfusion were excluded. A sensitivity analysis restricted to patients with directly measured PaO_2_ demonstrated similar SOFA predictive performance (AUC 0.845 vs. 0.847 for the full cohort), confirming the robustness of the estimation approach.

Detailed definitions of all score components, measurement times, data sources, and missing data handling are provided in Supporting Table 1.

All qSOFA and SOFA components were calculated using data obtained at emergency department presentation (index time point). Post‐admission or repeated measurements within 24 h were not used, in order to avoid incorporation of postbaseline information and to prevent inflation of predictive performance.

### 2.5. Outcome Measures

The primary endpoint was 28‐day all‐cause mortality. Survival status was initially determined from electronic medical records, including inpatient records, discharge summaries, and documented postdischarge encounters. For patients whose 28‐day survival status could not be confirmed through hospital records, structured telephone follow‐up was conducted using a standardized protocol between Day 28 and Day 35 after the index emergency department presentation. Survival status was verified through direct patient contact or by interviewing a first‐degree relative or primary caregiver. To reduce misclassification bias, at least two patient identifiers were confirmed, and all telephone‐reported outcomes were cross‐checked against hospital records where available. This structured follow‐up procedure ensured high reliability of mortality ascertainment, with no patients lost to follow‐up after repeated contact attempts.

The patient was contacted directly whenever possible; when the patient was unable to respond, a family member or primary caregiver familiar with the patient’s outcome was interviewed. Up to three telephone attempts were made on different days and at different times. The caller verified the patient’s identity using at least two identifiers and recorded survival status, date of death when applicable, and the source of information on a standardized follow‐up form. Death from any cause within 28 days of the index emergency department presentation was classified as 28‐day mortality. Patients whose survival status remained unknown after three unsuccessful contact attempts were classified as lost to follow‐up and were excluded from the primary outcome analysis. Secondary endpoints comprised (1) in‐hospital mortality; (2) ICU admission from the emergency department; (3) ICU length of stay; (4) hospital length of stay; (5) mechanical ventilation requirement within 48 h; and (6) vasopressor support duration.

### 2.6. Statistical Analysis

Statistical analyses were performed using SPSS Version 26.0 (IBM Corp., Armonk, NY, USA) and MedCalc Version 20.0 (MedCalc Software Ltd., Ostend, Belgium). A two‐sided *p* value < 0.05 was considered statistically significant. The distribution of continuous variables was assessed using the Shapiro–Wilk test. Normally distributed variables are presented as mean ± standard deviation, non‐normally distributed variables as median with interquartile range, and categorical variables as frequencies and percentages. Comparisons between survivors and nonsurvivors were performed using the independent‐samples *t* test or Mann–Whitney U test for continuous variables and the chi‐square test or Fisher’s exact test for categorical variables, as appropriate.

The discriminative performance of qSOFA and SOFA for 28‐day mortality and ICU admission was evaluated using receiver operating characteristic curves. Areas under the curve, sensitivity, specificity, positive predictive value, negative predictive value, and likelihood ratios were calculated at optimal cutoff values determined using the Youden index. Correlated AUCs were compared using the DeLong method. Variables associated with 28‐day mortality in univariable logistic regression analyses at *p* < 0.10 were considered for inclusion in a backward‐elimination multivariable logistic regression model. Results are reported as odds ratios with 95% confidence intervals. Kaplan–Meier curves and log‐rank tests were used for survival comparisons, and model calibration was assessed using calibration plots, calibration slope, intercept, and the Brier score, in addition to the Hosmer–Lemeshow test. A combined qSOFA–lactate model was constructed by adding serum lactate as a continuous variable to qSOFA and was compared with qSOFA alone and SOFA.

The primary analyses were conducted in the complete‐case cohort of 486 patients. Of the 527 patients who otherwise met the clinical eligibility criteria, 41 (7.8%) had at least one missing SOFA component. Missing data involved bilirubin in 21 patients (4.0%), PaO_2_/FiO_2_ in 18 (3.4%), creatinine or urine‐output data in 9 (1.7%), platelet count in 6 (1.1%), and Glasgow Coma Scale documentation in 3 (0.6%); some patients had more than one missing component.

As a sensitivity analysis, missing SOFA components were imputed using multiple imputation by chained equations under a missing‐at‐random (MAR) assumption, which is considered reasonable in this cohort because missing laboratory values were primarily driven by logistical testing delays and emergency department workflow constraints rather than patient outcomes or severity alone. This assumption was further supported by the similarity of baseline qSOFA and demographic variables between complete and incomplete cases. The imputation model included all SOFA and qSOFA components, age, sex, body mass index, infection source, comorbidities, heart rate, respiratory rate, systolic blood pressure, mean arterial pressure, oxygen saturation, lactate, procalcitonin, vasopressor use, mechanical ventilation, and 28‐day mortality status. Ten imputed datasets were generated, with 20 iterations per dataset. Continuous variables were imputed using predictive mean matching, binary variables using logistic regression, and categorical variables using multinomial logistic regression. The ROC and multivariable logistic‐regression analyses specified above were repeated in each imputed dataset. Regression coefficients and standard errors were combined using Rubin’s rules, and pooled AUC estimates were obtained after logit transformation. Results from the multiple‐imputation cohort of 527 patients were compared with those from the 486‐patient complete‐case cohort.

To reduce potential overfitting and to evaluate model stability, internal validation was performed using bootstrap resampling with 1000 iterations. The optimism‐corrected AUC for SOFA was 0.842 (95% CI 0.807–0.878), compared with the apparent AUC of 0.847, indicating minimal optimism (Δ = 0.005). For qSOFA, the bootstrap‐corrected AUC was 0.719. In addition, 10‐fold cross‐validation yielded consistent results (SOFA AUC 0.839; qSOFA AUC 0.716), confirming model robustness.

An exploratory subgroup analysis was conducted according to infection source. Because several categories contained relatively few patients, infection sources were grouped as pulmonary, abdominal, urinary, and other infections; the other‐infection category included soft‐tissue, bloodstream, and other or unknown sources. Within each subgroup, the AUCs of SOFA and qSOFA for 28‐day mortality were estimated and compared using the DeLong method. Logistic‐regression models containing score‐by‐infection‐source interaction terms were used to assess whether the prognostic association of each score varied across infection‐source categories. These analyses were considered exploratory because the study was not specifically powered for subgroup comparisons.

## 3. Results

### 3.1. Patient Characteristics

**FIGURE 1:**
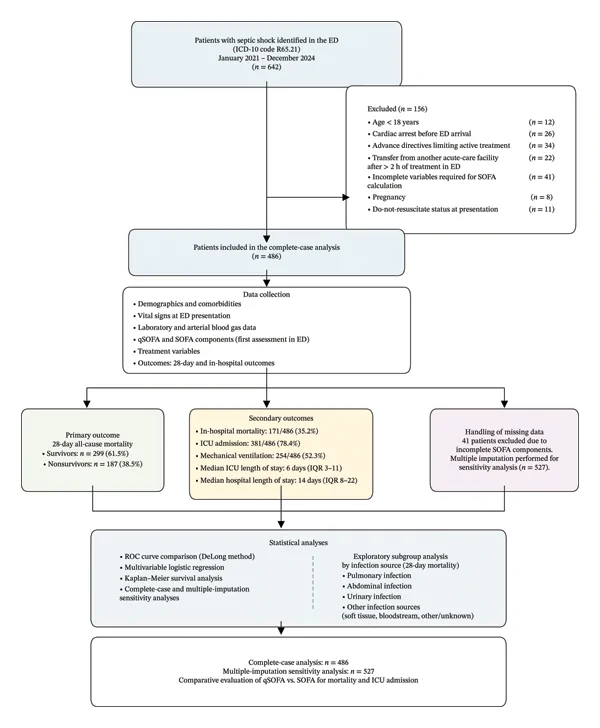
Flow diagram of patient identification, exclusion, enrollment, data collection, and outcome assessment.

Of the 642 patients initially identified, 115 were excluded because of clinical exclusion criteria, leaving 527 clinically eligible patients. Among these, 486 had complete data for both SOFA and qSOFA calculations and constituted the primary complete‐case cohort. The remaining 41 patients had one or more missing SOFA components and were included only in the multiple‐imputation sensitivity analysis (Figure 1). Compared with included patients, patients excluded from the complete‐case analysis because of incomplete SOFA data had higher baseline lactate levels (median, 5.2 vs. 4.2 mmol/L; *p* = 0.030), whereas age, sex, and qSOFA score did not differ significantly between the groups. Detailed comparisons are presented in Supporting Table 2. Demographic profile: mean age 64.8 ± 14.2 years; male predominance (285/486, 58.6%). The distribution of infection sources did not differ significantly between survivors and nonsurvivors (*p* = 0.124). Pulmonary infections accounted for 38.5%, abdominal 26.7%, urinary 18.1%, soft tissue 7.8%, bloodstream 4.9%, and other/unknown 3.9% (Table 1).

**TABLE 1:** Baseline demographics and clinical characteristics of the study population.

Variable	Total (*n* = 486)	Survivors (*n* = 299)	Nonsurvivors (*n* = 187)	*p*‐value
Demographics				
Age (years), mean ± SD	64.8 ± 14.2	61.5 ± 13.8	70.1 ± 13.2	< 0.001^∗^
Male, *n* (%)	285 (58.6)	172 (57.5)	113 (60.4)	0.521
BMI (kg/m^2^), mean ± SD	24.2 ± 4.6	24.5 ± 4.4	23.7 ± 4.9	0.068
Comorbidities, *n* (%)				
Diabetes mellitus	142 (29.2)	78 (26.1)	64 (34.2)	0.052
Hypertension	198 (40.7)	118 (39.5)	80 (42.8)	0.462
Chronic kidney disease	86 (17.7)	42 (14.0)	44 (23.5)	0.007^∗^
COPD	72 (14.8)	38 (12.7)	34 (18.2)	0.094
Malignancy	94 (19.3)	48 (16.1)	46 (24.6)	0.018^∗^
Immunosuppression	68 (14.0)	32 (10.7)	36 (19.3)	0.008^∗^
Infection source, *n* (%)				0.124
Pulmonary	187 (38.5)	108 (36.1)	79 (42.2)	
Abdominal	130 (26.7)	84 (28.1)	46 (24.6)	
Urinary	88 (18.1)	60 (20.1)	28 (15.0)	
Soft tissue	38 (7.8)	25 (8.4)	13 (7.0)	
Bloodstream	24 (4.9)	12 (4.0)	12 (6.4)	
Other/unknown	19 (3.9)	10 (3.3)	9 (4.8)	
Vital signs at presentation				
Heart rate (bpm), mean ± SD	112.4 ± 22.8	108.6 ± 21.5	118.5 ± 23.6	< 0.001^∗^
Respiratory rate (/min), mean ± SD	26.8 ± 7.4	24.9 ± 6.8	29.8 ± 7.5	< 0.001^∗^
SBP (mmHg), mean ± SD	86.2 ± 18.4	89.8 ± 17.2	80.4 ± 18.9	< 0.001^∗^
MAP (mmHg), mean ± SD	62.4 ± 12.6	65.2 ± 11.8	57.9 ± 12.5	< 0.001^∗^
Temperature (°C), mean ± SD	38.2 ± 1.4	38.3 ± 1.3	38.0 ± 1.5	0.024^∗^
GCS, median (IQR)	13 (10–15)	14 (12–15)	11 (8–14)	< 0.001^∗^
Laboratory values				
WBC (× 10^9^/L), mean ± SD	16.8 ± 9.4	15.9 ± 8.6	18.2 ± 10.4	0.012^∗^
Platelet (× 10^9^/L), mean ± SD	168.4 ± 98.6	186.2 ± 92.4	139.8 ± 101.2	< 0.001^∗^
Creatinine (μmol/L), median (IQR)	142 (86–228)	124 (78–195)	178 (108–286)	< 0.001^∗^
Bilirubin (μmol/L), median (IQR)	24.6 (14.2–48.5)	21.2 (12.8–38.6)	32.4 (18.5–68.2)	< 0.001^∗^
Lactate (mmol/L), mean ± SD	4.8 ± 2.6	3.8 ± 2.0	6.4 ± 2.8	< 0.001^∗^
Procalcitonin (ng/mL), median (IQR)	8.6 (2.4–28.5)	6.2 (1.8–18.4)	14.8 (4.6–42.6)	< 0.001^∗^
Scoring systems				
qSOFA score, mean ± SD	2.1 ± 0.8	1.8 ± 0.7	2.5 ± 0.6	< 0.001^∗^
qSOFA ≥ 2, *n* (%)	342 (70.4)	176 (58.9)	166 (88.8)	< 0.001^∗^
SOFA score, mean ± SD	9.4 ± 3.8	7.6 ± 3.2	12.3 ± 3.1	< 0.001^∗^

Abbreviations: BMI, body mass index; COPD, chronic obstructive pulmonary disease; GCS, Glasgow Coma Scale; IQR, interquartile range; MAP, mean arterial pressure; SBP, systolic blood pressure; SD, standard deviation; WBC, white blood cell count.

^∗^ *p* < 0.05 indicates statistical significance.

Several clinically significant differences emerged between survivors and nonsurvivors. Age demonstrated meaningful disparity (nonsurvivors: 70.1 ± 13.2 vs. survivors: 61.5 ± 13.8 years, Δ8.6 years, *p* < 0.001). Comorbidity prevalence was markedly higher among nonsurvivors: chronic kidney disease (23.5% vs. 14.0%, Δ9.5% points, *p* = 0.007), malignancy (24.6% vs. 16.1%, Δ8.5% points, *p* = 0.018), and immunosuppression (19.3% vs. 10.7%, Δ8.6% points, *p* = 0.008). Hemodynamic parameters and respiratory rates were significantly worse in nonsurvivors, with notably lower GCS scores and more pronounced laboratory abnormalities—particularly lactate (6.4 ± 2.8 vs. 3.8 ± 2.0 mmol/L, Δ2.6 mmol/L, *p* < 0.001) and procalcitonin elevations.

### 3.2. Outcomes

Twenty‐eight‐day survival status was obtained from electronic medical records for 414 patients (85.2%) and through telephone follow‐up for 72 patients (14.8%). Telephone follow‐up was successfully completed for all 72 patients requiring additional outcome ascertainment, and no patients were lost to 28‐day follow‐up. Overall 28‐day mortality was 38.5% (187/486), while in‐hospital mortality was 35.2% (171/486). ICU admission occurred in 78.4% of cases (381/486), with a median ICU stay of 6 days (IQR: 3–12). Mechanical ventilation was required by 52.3% (254/486), and median hospitalization duration was 14 days (IQR: 7–24).

### 3.3. Comparative Diagnostic Performance of qSOFA and SOFA

**FIGURE 2:**
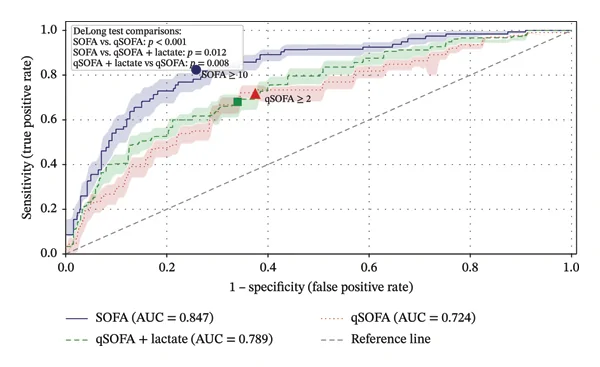
Receiver operating characteristic curves comparing qSOFA, SOFA, and combined qSOFA–lactate model discriminative ability for 28‐day mortality prediction in septic shock patients.

**FIGURE 3:**
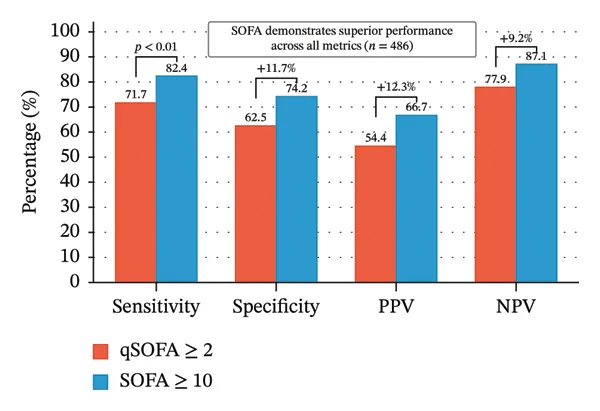
Comparative bar chart depicting sensitivity, specificity, PPV, and NPV for qSOFA ≥ 2 versus SOFA ≥ 10 in 28‐day mortality prediction.

ROC curve analysis revealed that SOFA had substantially better discriminative ability than qSOFA for predicting 28‐day mortality (Table 2, Figure 2). The AUC difference was highly significant: 0.847 (95% CI 0.812–0.882) for SOFA vs. 0.724 (95% CI 0.679–0.769) for qSOFA, Δ = 0.123 (*p* < 0.001, DeLong test). At optimal thresholds, SOFA (≥ 10) yielded 82.4% sensitivity and 74.2% specificity, whereas qSOFA (≥ 2) yielded 71.7% sensitivity and 62.5% specificity (Δ −10.7% and −11.7% points, respectively). The lactate‐augmented qSOFA model achieved intermediate discrimination (AUC 0.789, 95% CI 0.748–0.830), significantly outperforming qSOFA alone (*p* = 0.008) but remaining inferior to SOFA (*p* = 0.012). Figure 3 visualizes the comparative diagnostic metrics.

**TABLE 2:** Diagnostic performance of scoring systems for 28‐day mortality prediction.

Parameter	qSOFA	SOFA	qSOFA + lactate	*p*‐value (qSOFA vs. SOFA)
AUC (95% CI)	0.724 (0.679–0.769)	0.847 (0.812–0.882)	0.789 (0.748–0.830)	< 0.001^∗^
Optimal cutoff	≥ 2	≥ 10	—	—
Sensitivity (%)	71.7	82.4	76.5	—
Specificity (%)	62.5	74.2	68.9	—
PPV (%)	54.4	66.7	60.8	—
NPV (%)	77.9	87.1	82.4	—
+LR	1.91	3.19	2.46	—
−LR	0.45	0.24	0.34	—
Youden index	0.342	0.566	0.454	—

*Note:* +LR: positive likelihood ratio; −LR: negative likelihood ratio.

Abbreviations: AUC, area under the curve; CI, confidence interval; NPV, negative predictive value; PPV, positive predictive value.

^∗^DeLong test.

### 3.4. Sensitivity Analysis Using Multiple Imputation

The multiple‐imputation sensitivity analysis included all 527 clinically eligible patients, including the 41 patients with one or more missing SOFA components. SOFA remained more discriminative than qSOFA for 28‐day mortality in the imputed cohort. The pooled AUC was 0.842 (95% CI, 0.808–0.876) for SOFA and 0.719 (95% CI, 0.676–0.762) for qSOFA, with a pooled AUC difference of 0.123 (*p* < 0.001). These estimates were similar to those obtained in the complete‐case cohort, in which the AUCs were 0.847 (95% CI, 0.812–0.882) and 0.724 (95% CI, 0.679–0.769), respectively.

In the pooled multivariable logistic regression analysis, SOFA remained independently associated with 28‐day mortality (pooled OR per 1‐point increase, 1.40; 95% CI, 1.29–1.52; *p* < 0.001), consistent with the complete‐case estimate (OR, 1.42; 95% CI, 1.31–1.54; *p* < 0.001). The direction and magnitude of associations for age, lactate, chronic kidney disease, immunosuppression, time to antibiotics, and mechanical ventilation were also materially unchanged. These findings indicate that the principal results were not substantially altered by the handling of missing SOFA variables (Supporting Table 3).

### 3.5. Exploratory Subgroup Analysis by Infection Source

SOFA demonstrated numerically higher discrimination than qSOFA across all infection‐source subgroups. Among patients with pulmonary infection, the AUC was 0.851 for SOFA and 0.728 for qSOFA, with an AUC difference of 0.123 (*p* = 0.004). In the abdominal infection subgroup, the corresponding AUCs were 0.839 and 0.716, respectively (difference, 0.123; *p* = 0.018). Among patients with urinary infection, the AUCs were 0.861 for SOFA and 0.741 for qSOFA (difference, 0.120; *p* = 0.047). In patients with other infection sources, the AUCs were 0.825 and 0.702, respectively (difference, 0.123; *p* = 0.061).

There was no statistically significant interaction between infection‐source category and the prognostic effect of SOFA (*p* for interaction = 0.64) or qSOFA (*p* for interaction = 0.71), suggesting that the relative predictive performance of the two scores did not differ materially across infection sources (Supporting Table 4).

### 3.6. Predictive Performance for ICU Admission

SOFA also outperformed qSOFA for ICU admission prediction (AUC 0.798, 95% CI 0.758–0.838 vs. 0.686, 95% CI 0.638–0.734; Δ = 0.112, *p* < 0.001), with an optimal SOFA threshold of ≥ 8 (sensitivity 79.8%, specificity 68.6%).

### 3.7. Survival Analysis

**FIGURE 4:**
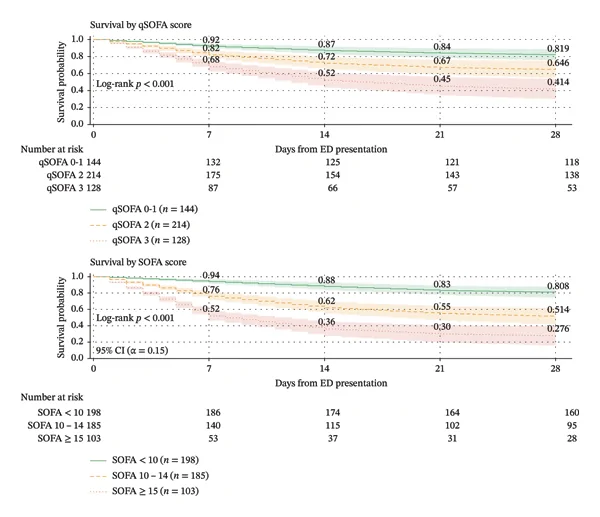
Kaplan–Meier survival curves stratified by qSOFA categories (0–1 vs. 2 vs. 3) and SOFA categories (< 10 vs. 10–14 vs. ≥ 15), illustrating dose–response relationships between score severity and survival probability.

Kaplan–Meier analysis demonstrated significant survival differences across both qSOFA and SOFA strata (log‐rank *p* < 0.001 for both; Figure 4), revealing a clear dose–response relationship between score severity and mortality risk. For qSOFA, 28‐day mortality rates were 18.1% (scores 0–1), 35.4% (score 2), and 58.6% (score 3), representing a 3.2‐fold increase from the lowest to highest stratum. For SOFA, the gradient was even steeper: 19.2% (< 10), 48.6% (10–14), and 72.4% (≥ 15), a 3.8‐fold increase across the severity spectrum.

### 3.8. Independent Predictors of 28‐Day Mortality

**FIGURE 5:**
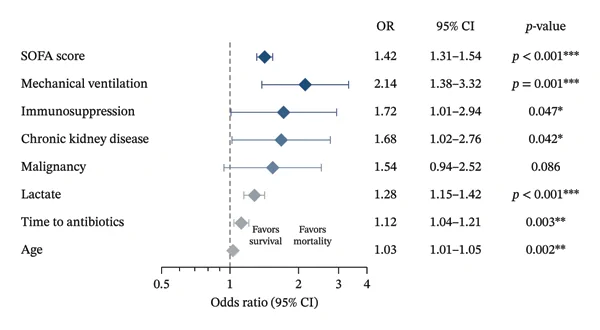
Forest plot displaying odds ratios and 95% confidence intervals for independent 28‐day mortality predictors identified through multivariate logistic regression.

Multivariable logistic regression identified several independent predictors of mortality (Table 3, Figure 5). The SOFA score was the strongest predictor (OR per 1‐point increase = 1.42, 95% CI 1.31–1.54, *p* < 0.001), corresponding to a 42% increase in mortality odds per point. Additional independent correlates included age (OR = 1.03 per year, *p* = 0.002), lactate (OR = 1.28 per mmol/L, *p* < 0.001), chronic kidney disease (OR = 1.68, *p* = 0.042), immunosuppression (OR = 1.72, *p* = 0.047), antibiotic delay (OR = 1.12 per hour, *p* = 0.003), and mechanical ventilation requirement (OR = 2.14, *p* = 0.001). Of note, qSOFA was excluded from the final model when SOFA was included, indicating that qSOFA provides little additional information beyond that captured by SOFA.

**TABLE 3:** Multivariate logistic regression analysis for 28‐day mortality prediction.

Variable	Odds ratio	95% CI	*p*‐value
SOFA score (per 1‐point increase)	1.42	1.31–1.54	< 0.001^∗^
Age (per 1‐year increase)	1.03	1.01–1.05	0.002^∗^
Lactate (per 1 mmol/L increase)	1.28	1.15–1.42	< 0.001^∗^
Chronic kidney disease	1.68	1.02–2.76	0.042^∗^
Malignancy	1.54	0.94–2.52	0.086
Immunosuppression	1.72	1.01–2.94	0.047^∗^
Time to antibiotics (per 1‐h delay)	1.12	1.04–1.21	0.003^∗^
Mechanical ventilation requirement	2.14	1.38–3.32	0.001^∗^

Abbreviation: CI, confidence interval.

^∗^ *p* < 0.05 indicates statistical significance.

### 3.9. Model Calibration

Hosmer–Lemeshow goodness‐of‐fit tests demonstrated acceptable calibration for both SOFA (*χ* ^2^ = 9.42, *p* = 0.308) and qSOFA (*χ* ^2^ = 7.86, *p* = 0.164), confirming satisfactory alignment between predicted and observed mortality across risk strata.

Calibration analysis demonstrated good agreement between predicted and observed outcomes. For SOFA, the calibration slope was 0.97 and the intercept was 0.02, with a Brier score of 0.184, indicating good overall accuracy. For qSOFA, the calibration slope was 0.91 with a Brier score of 0.221, suggesting slightly poorer calibration compared with SOFA.

## 4. Discussion

This head‐to‐head comparison of qSOFA and SOFA for prognostic assessment in emergency department patients with septic shock addresses a clinically consequential population in which risk stratification accuracy directly guides therapeutic decision‐making and resource allocation. Importantly, all included patients fulfilled the Sepsis‐3 definition of septic shock, ensuring a homogeneous high‐acuity population characterized by vasopressor‐dependent hypotension and elevated lactate despite adequate fluid resuscitation. qSOFA was originally developed as a rapid bedside tool for identifying patients with suspected infection who are at increased risk of adverse outcomes, rather than as a comprehensive prognostic model for patients with established septic shock according to Sepsis‐3 criteria. Because all patients in the present study had already met Sepsis‐3 criteria for septic shock, the study population represented a highly selected and severely ill cohort. In this context, the superior performance of SOFA, which incorporates multiple domains of organ dysfunction, may be partly inherent to the study design. Therefore, our findings should not be interpreted as evidence against the clinical utility of qSOFA as an early screening tool.

Although qSOFA and SOFA serve different clinical purposes, their comparison in this study is justified because both are widely used or referenced tools for risk stratification in suspected sepsis and septic shock. qSOFA represents a rapid bedside screening instrument, whereas SOFA provides a comprehensive organ dysfunction score. Comparing their prognostic performance in a homogeneous septic shock population allows evaluation of their relative utility in emergency department triage after diagnosis has been established.

The discriminative advantage of SOFA was substantial and statistically robust. The observed difference in AUC (0.847 vs. 0.724; Δ = 0.123) reflects a clinically meaningful gap in risk stratification. This translates to approximately 20 additional correctly predicted deaths among the 187 nonsurvivors. In their tertiary care center analysis, Mukherjee et al. similarly documented superior prognostic accuracy of SOFA over qSOFA in patients with suspected infection presenting to the emergency department [17]. Our results extend these observations specifically to established septic shock, where clinical stakes are highest and the value of comprehensive assessment appears most pronounced.

The mechanistic basis for SOFA’s superior discrimination lies in its comprehensive multiorgan evaluation, which captures the heterogeneous pathophysiology of septic shock. As Churpek et al. have noted, inflammatory cascades, microcirculatory disturbances, and cellular metabolic failure vary across patients depending on infection source, pathogen virulence, host factors, and treatment timing [18]. Askim et al. demonstrated that renal, hepatic, and coagulation dysfunction—captured by SOFA but not by qSOFA—may precede or develop independently of hemodynamic and neurological derangements, thereby providing incremental prognostic information [19]. Gonzalez del Castillo et al. further observed that SOFA’s continuous component scoring (0–4 points per component) enables more granular risk stratification than the dichotomous variables of qSOFA [20]. The dose–response mortality relationship from our survival analysis—19.2%, 48.6%, and 72.4% for SOFA strata < 10, 10–14, and ≥ 15, respectively—vividly illustrates this discriminative capacity.

Our finding that adding lactate to qSOFA significantly improved predictive performance (AUC from 0.724 to 0.789; Δ = 0.065) offers a practical strategy to enhance bedside screening. Saito et al. have emphasized that lactate reflects tissue hypoperfusion and anaerobic metabolism, a central pathophysiological feature of septic shock that qSOFA’s clinical variables alone inadequately capture [21]. As Hotchkiss et al. point out, lactate integrates information about global oxygen delivery‐consumption balance, thereby complementing the hemodynamic and neurological parameters of qSOFA [22]. Vincent and Moreno similarly demonstrated the superiority of lactate‐augmented qSOFA across diverse sepsis populations [23]. Nevertheless, even the combined qSOFA–lactate model remained significantly inferior to SOFA (AUC 0.789 vs. 0.847; *p* = 0.012). This finding aligns with the recent validation analysis by Ranzani et al., suggesting that lactate cannot fully compensate for comprehensive organ dysfunction evaluation [24].

Regarding infection source, the distribution did not differ significantly between survivors and nonsurvivors. However, this lack of statistical significance should not be interpreted as evidence that the infection source is irrelevant to prognosis. The *p*‐value may reflect limited statistical power for detecting such differences, particularly given the relatively small sample sizes in some source categories (e.g., bloodstream and soft‐tissue infections). Our exploratory subgroup analyses demonstrated that SOFA consistently outperformed qSOFA across all infection source groups, with no significant score‐by‐source interaction (*p* for interaction = 0.64 for SOFA and 0.71 for qSOFA). These findings suggest that the superior prognostic performance of SOFA over qSOFA is robust across different infection sources in this septic shock population.

These findings support a tiered risk stratification approach in the management of septic shock in the emergency department. As Chen et al. have proposed, qSOFA retains value as an initial screening tool that enables rapid identification of patients warranting urgent evaluation. Its simplicity and immediate availability—requiring no laboratory results—represent practical advantages [25]. However, our data align with the conclusion of Liu et al. that qSOFA should not serve as the definitive prognostic instrument for treatment decisions in established septic shock, a setting in which comprehensive SOFA assessment provides meaningfully superior risk stratification [26]. In their update on Surviving Sepsis Campaign research priorities, De Backer et al. similarly emphasized the need for rapid SOFA calculation workflows once initial laboratory results become available [27]. Particular attention should be directed toward patients with discordant qSOFA and SOFA assessments, who may face an elevated risk of misclassification, as highlighted by Li et al. in their multicenter analysis [28]. This design ensures that both scoring systems reflect real‐time emergency department decision‐making conditions and supports clinical applicability for early triage. However, this setting also reflects a high‐volume emergency department environment where rapid triage and early risk stratification are critical, thereby enhancing the relevance of our findings for real‐world emergency decision‐making.

Several methodological limitations should be considered when interpreting these findings. As a single‐center retrospective study conducted in a tertiary emergency department, external generalizability may be limited. Patient characteristics, resource availability, and clinical workflows may differ across institutions, particularly in community hospitals or resource‐limited settings. (1) The retrospective, single‐center design inherently limits causal inference and generalizability; unmeasured confounding and institutional practice patterns may have influenced outcomes, and findings may not extend to community hospitals or populations with different demographic or infection profiles. (2) Requiring complete laboratory data for SOFA calculation introduced selection bias, because patients with missing values (e.g., arterial blood gas, bilirubin, platelets) were excluded from the primary analysis, and these patients may have differed systematically in illness severity or clinical management from those with complete data. Although multiple imputation yielded consistent results, residual bias cannot be excluded. (3) The use of estimated PaO_2_/FiO_2_ ratios in 16.1% of patients, while validated, introduces nondifferential measurement error that could modestly attenuate the observed discriminative performance of SOFA. (4) We assessed qSOFA and SOFA only at presentation or within the first 24 h, without capturing dynamic changes over time. As Ruan et al. have demonstrated, serial score assessments may provide additional prognostic information beyond baseline values, and our static assessment may therefore underestimate the true predictive capacity of both instruments [29]. (5) The optimal cutoff values we identified (e.g., SOFA ≥ 10 for mortality) were derived from a single cohort and lack external validation. As recommended by Wang et al. in their recent meta‐analysis, such thresholds require validation in independent populations before widespread clinical implementation [30]. (6) The exploratory subgroup analyses by infection source were not powered for formal interaction testing, and the small sample sizes in some categories (e.g., bloodstream infections) increase the risk of type II error and unstable estimates.

## 5. Conclusion

In this cohort of patients with established septic shock according to Sepsis‐3 criteria, SOFA demonstrated superior mortality discrimination compared with qSOFA in patients with septic shock. These findings were robust across sensitivity analyses and supported by TRIPOD‐compliant model reporting and PROBAST‐based risk of bias assessment. These findings pertain to prognostic assessment after septic shock has been diagnosed and may not be directly generalizable to the use of qSOFA as an early bedside screening tool in broader populations with suspected infection. Although qSOFA offers clear advantages in simplicity and immediate bedside applicability, its sensitivity limitation—an observed deficit of 10.7% points relative to SOFA in this study—suggests that it may not be sufficient as a standalone prognostic instrument in this critically ill population. SOFA may remain the preferred tool for comprehensive risk stratification, whereas qSOFA could serve as an initial screening measure whose utility might be enhanced by the addition of serum lactate.

## Author Contributions

We declare that all the listed authors have participated actively in the study and all meet the requirements for authorship. Xue Liu & Huajie Zhang designed the study, managed the literature searches, and wrote the manuscript. Qirong He undertook the data acquisition and analysis. All authors reviewed the manuscript.

## Funding

This research did not receive any specific funding.

## Ethics Statement

Institutional Review Board approval was obtained from the Yibin First People’s Hospital (Approval number: SYBFL1250551, with approved date 2025/12/25), with informed consent requirements waived given the retrospective design. All procedures adhered to Declaration of Helsinki principles, with reporting following STROBE guidelines for observational investigations.

## Conflicts of Interest

The authors declare no conflicts of interest.

## Supporting Information

Additional supporting information can be found online in the Supporting Information section.

## Supporting information


**Supporting Information** Supporting Figure 1. PROBAST risk‐of‐bias assessment for prediction models of 28‐day mortality in emergency department patients with septic shock, summarizing risk of bias across the participants, predictors, outcome, and analysis domains. Supporting Table 1. Definitions, measurement timing, data sources, and missing‐data handling for SOFA and qSOFA score components. Supporting Table 2. Comparison of baseline characteristics between patients included in the complete‐case analysis and patients excluded because of incomplete data. Supporting Table 3. Comparison of complete‐case and multiple‐imputation analyses for 28‐day mortality prediction. Supporting Table 4. Subgroup comparison of SOFA and qSOFA performance for 28‐day mortality according to infection source. STROBE Checklist. Completed the STROBE statement checklist for reporting this retrospective cohort study, with the location of each applicable reporting item indicated in the manuscript.

## Data Availability

The data that support the findings of this study are available from the corresponding author upon reasonable request.
