# Supplementary material for: Comparative Prognostic Performance of qSOFA and SOFA in Emergency Department Patients With Established Septic Shock
Source: Emerg Med Int. 2026 Aug 27;2026:1902660. doi: 10.1155/emmi/1902660 (PMC13519888; doi:10.1155/emmi/1902660)
Supplement: Supplementary file 1 [file EMMI-2026-1902660-s001.zip › STROBE_Checklist_PageFilled.docx]

# STROBE Statement — Checklist for Cross-sectional Studies

| Item No. | Recommendation | Page No. |
| --- | --- | --- |
| 1 | Title and abstract: Indicate study design in title or abstract | 1-2 |
| 2 | Background/rationale: Explain scientific background and rationale | 2-4 |
| 3 | Objectives: State specific objectives | 4 |
| 4 | Study design: Present key elements of study design early | 4-5 |
| 5 | Setting: Describe setting, locations, and relevant dates | 4-5 |
| 6 | Participants: Eligibility criteria and selection process | 5-6 |
| 7 | Variables: Clearly define outcomes, exposures, predictors | 6-8 |
| 8 | Data sources/measurement: Describe methods of assessment | 6-8 |
| 9 | Bias: Describe efforts to address potential sources of bias | 10-11,26-27 |
| 10 | Study size: Explain how study size was determined | 5-6 |
| 11 | Quantitative variables: Explain handling of quantitative variables | 10 |
| 12 | Statistical methods: Describe all statistical methods | 10-11 |
| 13 | Participants flow: Report numbers at each stage | 12-15 |
| 14 | Descriptive data: Report characteristics of study population | 12-14 |
| 15 | Outcome data: Report outcome events or summaries | 16 |
| 16 | Main results: Provide unadjusted and adjusted estimates | 16-18 |
| 17 | Other analyses: Report subgroup and sensitivity analyses | 18-20 |
| 18 | Key results: Summarize key findings in discussion | 22-23 |
| 19 | Limitations: Discuss study limitations | 26 |
| 20 | Interpretation: Provide cautious interpretation | 23-25 |
| 21 | Generalisability: Discuss external validity | 25-26 |
| 22 | Funding: State funding source and role | 28 |
