# Supplementary material for: Comparative Prognostic Performance of qSOFA and SOFA in Emergency Department Patients With Established Septic Shock
Source: Emerg Med Int. 2026 Aug 27;2026:1902660. doi: 10.1155/emmi/1902660 (PMC13519888; doi:10.1155/emmi/1902660)
Supplement: Supplementary file 1 [file EMMI-2026-1902660-s001.zip › Supplementary Figure 1_editable.pdf]

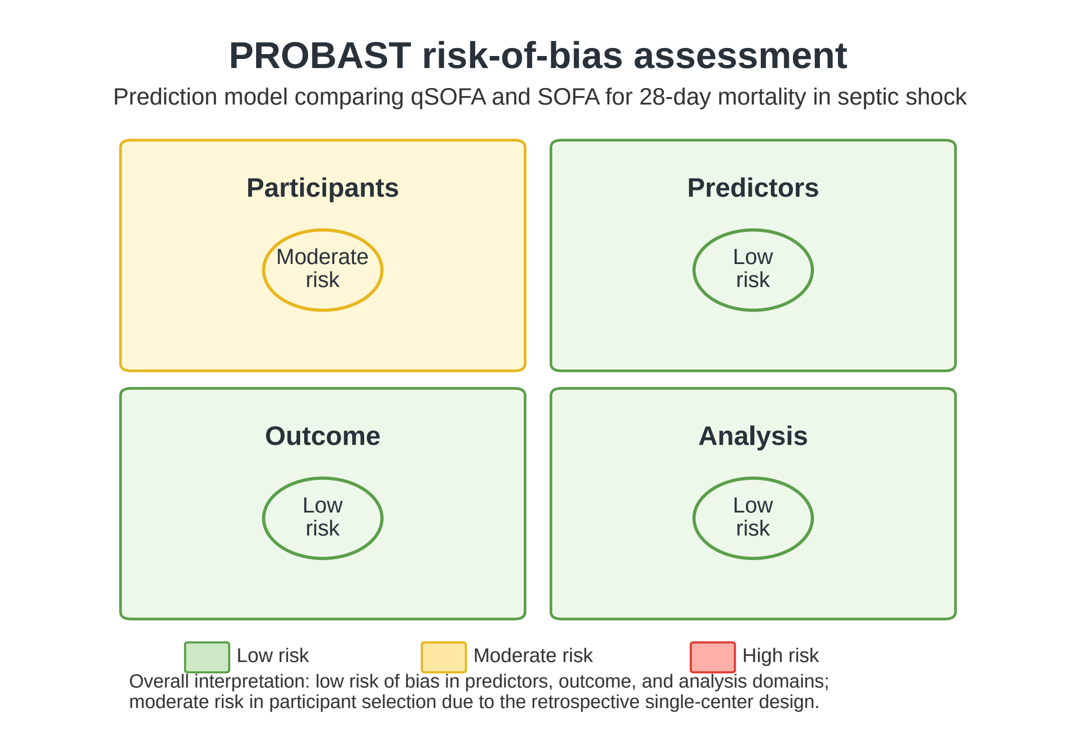
PROBAST risk-of-bias assessment
Prediction model comparing qSOFA and SOFA for 28-day mortality in septic shock
Participants
Moderate risk
Predictors
Low risk
Outcome
Low risk
Analysis
Low risk
Low risk
Moderate risk
High risk
Overall interpretation: low risk of bias in predictors, outcome, and analysis domains;
moderate risk in participant selection due to the retrospective single-center design.
