# Supplementary material for: Comparative Prognostic Performance of qSOFA and SOFA in Emergency Department Patients With Established Septic Shock
Source: Emerg Med Int. 2026 Aug 27;2026:1902660. doi: 10.1155/emmi/1902660 (PMC13519888; doi:10.1155/emmi/1902660)
Supplement: Supplementary file 1 [file EMMI-2026-1902660-s001.zip › Supplementary Figure 1 legends.doc]

**Supplementary Figure 1**. PROBAST risk-of-bias assessment for prediction models of 28-day mortality in emergency department patients with septic shock.
