# Supplementary material for: Comparative Prognostic Performance of qSOFA and SOFA in Emergency Department Patients With Established Septic Shock
Source: Emerg Med Int. 2026 Aug 27;2026:1902660. doi: 10.1155/emmi/1902660 (PMC13519888; doi:10.1155/emmi/1902660)
Supplement: Supplementary file 1 [file EMMI-2026-1902660-s001.zip › Supplementary Table 1.doc]

**Supplementary Table 1**. Definitions, measurement timing, data sources, and missing data handling for SOFA and qSOFA score components

| **Variable** | **Definition** | **Measurement time** | **Data source** | **Missing value handling** |
| --- | --- | --- | --- | --- |
| PaO₂/FiO₂ | Arterial oxygen / inspired O₂ fraction. If ABG unavailable, estimated using SpO₂/FiO₂ with Rice et al. (2007) formula: PaO₂/FiO₂ = 64 + 0.84 × (SpO₂/FiO₂) | First 24h, worst value | ABG or estimated from SpO₂ | Use estimated value if ABG missing; restricted to SpO₂ 80–97%; excluded if poor perfusion |
| Platelet | Platelet count (×10⁹/L) | First 24h, worst value | Lab test | N/A |
| Bilirubin | Serum total bilirubin (μmol/L) | First 24h, worst value | Lab test | N/A |
| MAP | Mean arterial pressure (mmHg) | First 24h, worst value | Vital signs | N/A |
| Vasopressor dose | Norepinephrine μg/kg/min equivalent | First 24h, peak value | EMR medication record | N/A |
| GCS | Glasgow Coma Scale | First 24h, lowest value | Clinical record | N/A |
| Creatinine | Serum creatinine (μmol/L) | First 24h, peak value | Lab test | N/A |
| Urine output | mL/kg/h | First 24h, lowest 6h block | Nursing record | N/A |
| qSOFA | 3-point clinical score | First 24h, worst value | Clinical assessment | N/A |
