# Supplementary material for: Comparative Prognostic Performance of qSOFA and SOFA in Emergency Department Patients With Established Septic Shock
Source: Emerg Med Int. 2026 Aug 27;2026:1902660. doi: 10.1155/emmi/1902660 (PMC13519888; doi:10.1155/emmi/1902660)
Supplement: Supplementary file 1 [file EMMI-2026-1902660-s001.zip › Supplementary Table 2.doc]

### Supplementary Table 2. Comparison of baseline characteristics between included patients and patients excluded because of incomplete data

| **Variable** | **Included (n=486)** | **Excluded (n=41)** | **p-value** |
| --- | --- | --- | --- |
| Age, mean±SD | 64.8±14.2 | 66.1±15.0 | 0.48 |
| Male, n (%) | 285 (58.6) | 24 (58.5) | 0.99 |
| Lactate, median (IQR) | 4.2 (3.0–6.5) | 5.2 (3.5–7.0) | 0.03* |
| qSOFA, mean±SD | 2.1±0.8 | 2.2±0.9 | 0.31 |
| SOFA, mean±SD | 9.4±3.8 | NA | NA |
