# Supplementary material for: Comparative Prognostic Performance of qSOFA and SOFA in Emergency Department Patients With Established Septic Shock
Source: Emerg Med Int. 2026 Aug 27;2026:1902660. doi: 10.1155/emmi/1902660 (PMC13519888; doi:10.1155/emmi/1902660)
Supplement: Supplementary file 1 [file EMMI-2026-1902660-s001.zip › Supplementary Table 3.doc]

**Supplementary Table 3.** Comparison of complete-case and multiple-imputation analyses for 28-day mortality prediction

| **Parameter** | **Complete-case analysis (n=486)** | **Multiple-imputation analysis (n=527)** |
| --- | --- | --- |
| SOFA AUC (95% CI) | 0.847 (0.812–0.882) | 0.842 (0.808–0.876) |
| qSOFA AUC (95% CI) | 0.724 (0.679–0.769) | 0.719 (0.676–0.762) |
| Difference in AUC | 0.123 | 0.123 |
| p-value for AUC comparison | <0.001 | <0.001 |
| SOFA OR per point (95% CI) | 1.42 (1.31–1.54) | 1.40 (1.29–1.52) |
| SOFA p-value | <0.001 | <0.001 |
| Age OR per year (95% CI) | 1.03 (1.01–1.05) | 1.03 (1.01–1.05) |
| Lactate OR per mmol/L (95% CI) | 1.28 (1.15–1.42) | 1.26 (1.14–1.40) |
