# Supplementary material for: Comparative Prognostic Performance of qSOFA and SOFA in Emergency Department Patients With Established Septic Shock
Source: Emerg Med Int. 2026 Aug 27;2026:1902660. doi: 10.1155/emmi/1902660 (PMC13519888; doi:10.1155/emmi/1902660)
Supplement: Supplementary file 1 [file EMMI-2026-1902660-s001.zip › Supplementary Table 4.doc]

**Supplementary Table 4**. Subgroup comparison of SOFA and qSOFA performance for 28-day mortality according to infection source

| **Infection source** | **n** | **SOFA AUC (95% CI)** | **qSOFA AUC (95% CI)** | **AUC difference** | **p-value** | **p for interaction** |
| --- | --- | --- | --- | --- | --- | --- |
| Pulmonary | 187 | 0.851 (0.810–0.892) | 0.728 (0.680–0.776) | 0.123 | 0.004 | 0.64 |
| Abdominal | 130 | 0.839 (0.790–0.888) | 0.716 (0.660–0.772) | 0.123 | 0.018 | 0.64 |
| Urinary | 88 | 0.861 (0.807–0.915) | 0.741 (0.672–0.810) | 0.12 | 0.047 | 0.64 |
| Other | 81 | 0.825 (0.760–0.890) | 0.702 (0.630–0.774) | 0.123 | 0.061 | 0.64 |
